# Efficacy and safety of electromagnetic acupuncture using an electromagnetic therapy stimulator (Whata153) for the treatment of chronic low back pain: Study protocol for a single-center, parallel-arm, randomized clinical trial

**DOI:** 10.1097/MD.0000000000013047

**Published:** 2018-11-02

**Authors:** Seo Young Oh, Jae Hui Kang

**Affiliations:** Department of Acupuncture and Moxibustion Medicine, Cheonan Korean Medicine Hospital of Daejeon University, Chungcheongnamdo, Republic of Korea.

**Keywords:** chronic low back pain, electromagnetic acupuncture, electromagnetic stimulation, Oswestry Disability Index, randomized trial, study protocol, visual analog scale

## Abstract

**Background:**

Chronic low back pain is one of the major conditions causing serious personal and social difficulties in modern society. There are several noninvasive therapies for chronic low back pain; however, the effects of electromagnetic acupuncture have not been studied. Here, we describe the protocol for a study that will investigate the efficacy and safety of electromagnetic acupuncture for patients with chronic low back pain.

**Methods:**

The study has been designed as a double-blind, single-center, parallel-arm, sham-controlled, randomized clinical trial. A total of 104 patients with chronic low back pain who meet the criteria for selection and exclusion will be enrolled in a 1:1 ratio in an electromagnetic acupuncture group or a sham group. Sterilized disposable needles will be inserted at 6 acupoints, following which stimulation via an electromagnetic (Whata153) or a placebo (sham) stimulator will be applied. The 2 groups will receive a total of 6 treatment sessions over 3 weeks, with 1 follow-up visit within 3 days after the final treatment. The primary outcome will be the change in the visual analog scale (VAS) score for pain from baseline (visit 1, first treatment session) to the follow-up visit (visit 7, after treatment completion). The secondary outcomes will be as follows: changes in the VAS score for pain from baseline (visit 1) to visits 3 (third session) and 5 (fifth session); changes in the VAS score for pain at all assessment points from baseline (visit 1) to the follow-up visit (visit 7); changes in the Oswestry Disability Index (ODI) from visit 1 to visits 3, 5, and 7; and the change in ODI at all assessment points from visit 1 to visit 7.

**Discussion:**

The results of this trial are expected to provide important clinical information on the efficacy and safety of electromagnetic acupuncture for patients with chronic low back pain.

## 1 Introduction

Chronic low back pain is one of the major conditions causing severe personal and social difficulties in modern society. Nonspecific chronic low back pain refers to pain that lasts for at least 3 months without a pathological anatomical abnormality.^[1]^ Most Americans have experienced low back pain, and approximately a quarter of adults investigated have reported low back pain lasting for a minimum of 1 day in the past 3 months.^[2]^

The Guidelines for Chronic Back Pain of the American College of Medicine, published in 2017, emphasize the importance of nonpharmacological treatment. According to these guidelines, nonsteroidal anti-inflammatory drugs (NSAIDs) and muscle relaxants can be considered if symptoms do not improve after nonpharmacological treatment, such as acupuncture and physiotherapy. Tramadol or duloxeine can be considered if NSAIDs and muscle relaxants are ineffective. Therefore, the demand for oriental medicine treatment for chronic low back pain has been increasing of late.^[3]^

According to the Survey on the Use of Oriental Medicine by the Korean Ministry of Health and Welfare in 2017, 73.8% of Koreans overall use oriental medicine. Low back pain was the most common symptom, accounting for 52.7% of cases. More specifically, acupuncture, cupping, moxibustion, and oriental physiotherapy were used for 90.2%, 53.0%, 49.1%, and 40.2% cases, respectively.^[4]^

Low-frequency electroacupuncture is used to control pain and muscle tension. Many studies have shown that electroacupuncture has a better effect on low back pain than does conventional acupuncture.^[5–7]^ Some studies have also evaluated electromagnetic acupuncture, a less-commonly used modality, for muscle fatigue recovery, meridian potential changes, and activation of the autonomic nervous system.^[8,9]^

Electromagnetic stimulation is based on the following principle. When a magnetic field is applied to a human body, a new electric field and electric current are generated, and the ions electrically stimulate the surrounding nerves. The intensity of the electric field decreases as it penetrates the skin. The advantage of electromagnetic stimulation is that the loss of the electric field in the skin is much less than that associated with electrical stimulation. For example, using the same intensity of stimulation of the skin, electromagnetic stimulation induces an electric field that is 10 times higher than that induced by electrical stimulation in a nerve located within 40 mm of the skin.^[10]^ Because the intensity of stimulation required to produce the same effect is lower with electromagnetic stimulation than with electrical stimulation, patients experience less discomfort and pain and may experience more positive effects with the former than with the latter.

Electromagnetic acupuncture may therefore also be effective in relieving chronic back pain; however, few studies have validated this. When an electric current is applied to a coil that is wound into a cylindrical shape, a magnetic field is produced. When an acupuncture needle, which is a magnetic substance, is inserted into the coil, an electromagnet is formed and a stronger magnetic field is achieved.

We aim to investigate the efficacy and safety of electromagnetic acupuncture in patients with chronic low back pain and to collect the relevant data. Here, we describe the protocol for a parallel-arm, sham-controlled, randomized clinical trial for evaluating the efficacy and safety of electromagnetic acupuncture for patients with chronic low back pain. We intend to use the Whata153 (Medi Lab., Korea), a device that converts electric energy into magnetic energy, for electromagnetic stimulation.

## 2 Methods

### 2.1 Study design

This study has been designed as a double-blind, single-center, parallel-arm, sham-controlled, randomized clinical trial for investigating the efficacy and safety of electromagnetic acupuncture for treatment of patients with chronic low back pain.

A total of 104 patients with chronic low back pain who meet the criteria for selection and exclusion will be enrolled in a 1:1 ratio, with 52 patients included in an electromagnetic acupuncture group (EMA group) and 52 in a sham group.

### 2.2 Ethics approval

The study protocol has been approved by the Institutional Review Board of Cheonan Korean Medicine Hospital, Daejeon University (protocol number: M2015-01-1). The protocol was registered with Korean National Clinical Trial Registry CRIS (KCT0002915). The protocol accords with the Declaration of Helsinki.

### 2.3 Participants

#### 2.3.1 Inclusion criteria

The inclusion criteria are as follows: age, 19 to 70 years; presence of chronic low back pain for >3 months; a minimum pain intensity score of 40 mm on a 100-mm visual analog scale (VAS); ability of the participant to fully understand the trial procedure and the risks involved, communicate with the examiner, and comply with the protocol; and provision of written informed consent for participation.

#### 2.3.2 Exclusion criteria

The exclusion criteria are as follows: a history of spinal surgery; progressive neurological deficit or severe psychiatric or psychological disorders; serious spinal disorders, such as metastatic cancer, vertebral fracture, spinal infection, and inflammatory spondylitis; other contraindications for treatment, such as clotting disorders, use of anticoagulants, and seizure disorders; presence of a device that could be affected by electromagnetic fields, such as a pacemaker or a hearing aid; use of medications that could affect the trial results, such as corticosteroids and anticonvulsants, within the last week; pregnancy, lactation, or plans for conception; participation in other clinical trials; and ineligibility judged by a physician.

### 2.4 Procedures

**Figure 1:**
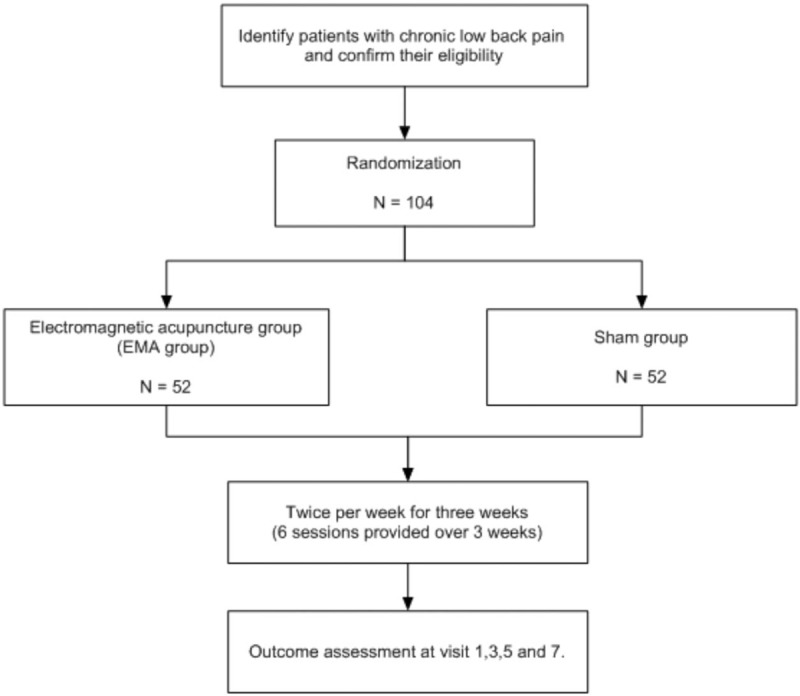
Study flow chart.

Patients with chronic low back pain will be recruited through advertisements on hospital bulletin boards and websites. Individuals interested in participating will be provided an explanation of the study until they have completely understood the risks and benefits. If they agree to participate, they will be asked to sign a consent form and undergo screening for eligibility. If they are found to be eligible, a study researcher will randomly allocate them to either the treatment or the sham group. The treatment procedure will be scheduled after randomization. The study flow chart is presented in Fig. 1.

### 2.5 Randomization and blinding procedures

The block randomization method will be used for randomization, at a 1:1 ratio, to ensure balanced distribution. The random assignment code will be calculated using a computer software program, and statisticians will assign codes according to the randomized lists. The randomization code will be sealed in an opaque envelope and stored at the hospital.

The patients and researchers performing treatment or assessing outcomes will be blinded to the treatment. To ensure blinding, needles for both groups will be manufactured in the same size and package. The Whata153 will be used for electromagnetic stimulation in the EMA group. In the sham group, a placebo stimulator with the same shape, sound, and machine motion will be used. The patients will not be able to identify the treatment, because the skin receives almost no sensory stimulus with electromagnetic stimulation.

Randomization and blinding are not disclosed to the investigator until the end of the clinical trial, except for disclosure to individual patients in an emergency.

### 2.6 Interventions

Both the EMA and sham groups will receive a total of 6 treatment sessions over 3 weeks, with 1 follow-up visit within 3 days after the last treatment (end of the clinical trial). The trial will be conducted under the same conditions for all participants, and the same practitioners will treat all participants. Other therapies and treatments associated with chronic low back pain will be excluded during the trial period. Use of medications other than those specified in the exclusion criteria (e.g., steroids, anticonvulsants) will be maintained. The acupuncture protocol to be used is based on the Standards for Reporting Interventions in Clinical Trials of Acupuncture (STRICTA) and is presented in Table 1.

**Table 1:**
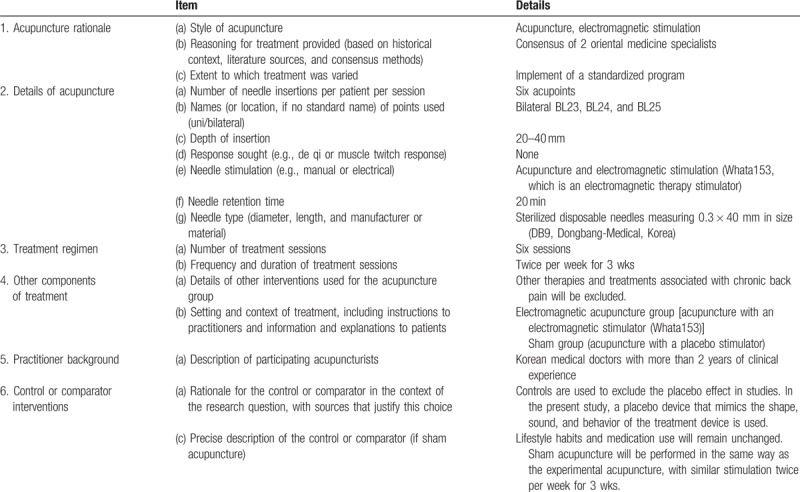
Electromagnetic acupuncture protocol, based on the Standards for Reporting Interventions in Clinical Trials of Acupuncture, for patients with chronic low back pain.

### 2.7 Acupuncture treatment

**Figure 2:**
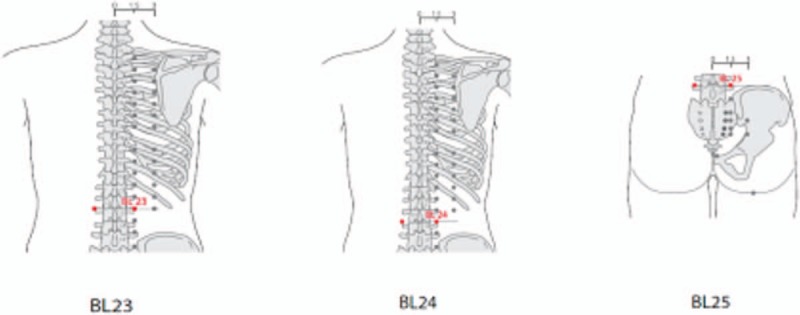
Location of acupoints used for treatment of chronic low back pain.

**Figure 3:**
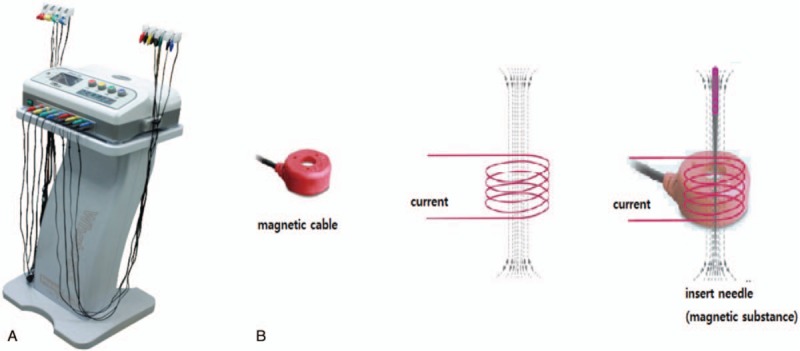
The Whata153 (A), which is an electromagnetic acupuncture device, and a diagram illustrating the process of electromagnetic acupuncture (B).

The bilateral BL23, BL24, and BL25 acupoints, which are frequently used for chronic low back pain, have been selected in accordance with previous studies^[11,12]^ (Fig. 2). Sterilized disposable needles measuring 0.3 × 40 mm in size (DB9; Dongbang-Medical, Sungnam, Korea) will be inserted at the 6 acupoints. After the needle is inserted, the Whata153 (EMA group) or a placebo stimulator (sham group) will be applied as follows. The ring in which the electromagnetic field is generated will be placed around the needle (Fig. 3). Stimulation lasting approximately 20 minutes in the E6 settling mode (500 Hz, interference wave) will then be applied.

### 2.8 Acupuncturists

Licensed Korean Medicine doctors with more than 2 years of clinical experience will perform the treatment procedures. All acupuncturists and researchers will strictly comply with the study protocol and complete the training program.

### 2.9 Data collection

During the screening period, the participants will complete a questionnaire regarding their sociodemographic characteristics, provide their medical history, undergo a physical examination in the lumbar region, and rate their pain using the VAS.

Personal information and data collected during the screening period will be shared and managed by the hospital. As the researchers are clinical, a data monitoring committee is not needed. The final trial dataset is accessible to statisticians and principal investigator.

The schedule for treatment and outcome measurements is presented in Table 2. Visits 1 to 6 will include the treatment sessions, while visit 7 will be the follow-up visit after treatment completion.

**Table 2:**
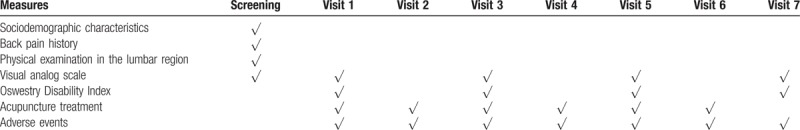
Schedule for treatment (electromagnetic acupuncture or placebo) and outcome measurements for patients with chronic low back pain.

### 2.10 Outcome measurements

The primary and secondary outcomes will be based on the VAS score for pain and the Oswestry Disability Index (ODI). The VAS has been shown to be a reliable tool for evaluating chronic pain.^[13]^ VAS for pain is a continuous scale with a horizontal or vertical line measuring 100 mm in length.^[14]^ Patients rate their current pain intensity from 0 (no pain) to 100 (worst pain imaginable).^[15]^ ODI was developed as a patient-oriented assessment tool and includes various questions that are answered by the patients themselves.^[16]^ The score is calculated as the total score/(number of questions provided by the author ∗ 5) ∗ 100.

#### 2.10.1 Primary outcomes

The change in the VAS score for pain from baseline (visit 1, first treatment session) to the follow-up visit after treatment completion (visit 7) will be assessed as the primary outcome.

#### 2.10.2 Secondary outcomes

The following will be assessed as secondary outcomes: changes in the VAS score for pain from baseline (visit 1) to visits 3 (third session) and 5 (fifth session); changes in the VAS score for pain at all assessment points from baseline (visit 1) to the follow-up visit (visit 7); changes in the ODI from visit 1 to visits 3, 5, and 7; and the change in ODI at all assessment points from visit 1 to visit 7.

### 2.11 Safety

The occurrence of any adverse events will be evaluated at each visit. Patients will be monitored for undesirable, unintended signs, symptoms, and illnesses. The number and percentage of participants who experience at least 1 adverse event will be calculated.

### 2.12 Withdrawal and dropout

Participants will be withdrawn from the trial if they do not meet the selection/exclusion criteria, withdraw consent, or conceive. The trial will be stopped if significant adverse events occur or if the patient is considered to be at risk upon further participation.

### 2.13 Sample size

The sample size will be calculated as per the results of previous trials on electroacupuncture therapy for chronic low back pain, because there are no available studies on electromagnetic acupuncture therapy. We assume that the effect of electromagnetic acupuncture would at least be equivalent to those of electroacupuncture; therefore, we will refer to the effect size of electroacupuncture therapy as conservative treatment for low back pain. In a previous study, the mean difference in the follow-up VAS score for pain was 0.87 between groups, with an estimated standard deviation of 1.48.^[17]^ The alpha level is 5% for both sides, and the study power is maintained at 80%. A total of 52 participants will thus be required in each group after considering an attrition rate of 10%.

### 2.14 Statistical analysis

Statistical analysis will be primarily based on the intent-to-treat principle, although per-protocol principles will also be checked. The missing values will be analyzed by the last observation carried forward method. The significance level will be set at *P* < .05, and the confidence interval at 95%. All statistical analyses will be performed using SPSS Statistics for Windows Version 20.0 (IBM Corp., Armonk, NY). There is no intermediate analysis of this study, and the final decision on end of trial is with principal investigator.

Differences in sociodemographic and baseline characteristics between the EMA and sham groups will be evaluated. For continuous data, Student *t* test or the Wilcoxon rank sum test will be performed. For categorical data, the Chi-square test or Fisher exact test will be used.

Changes in VAS and ODI scores from baseline to each assessment point will be assessed using Student *t* test or the Wilcoxon rank sum test, while those at all evaluation points from baseline to the follow-up visit will be assessed using repeated measures analysis of variance (RM-ANOVA). The incidence of adverse events will be investigated using the Chi-square test or Fisher exact test.

## 3 Discussion

Of late, nonpharmacological therapies for chronic low back pain have gained popularity as replacements for medication or surgery. In the field of oriental medicine, various treatments, such as acupuncture, electroacupuncture, pharmacopuncture, and acupotomy, are used for chronic low back pain.

Magnetic fields are used to treat various diseases.^[18]^ Use of electromagnetic stimulation therapy has been reported for inducing muscle relaxation,^[9]^ treating Bell palsy^[19]^ as well as musculoskeletal pain.^[20,21]^ To the best of our knowledge, this will be the first study on the use of electromagnetic acupuncture for the treatment of chronic low back pain.

This study design is expected to have some advantages. In order to verify the efficacy of electromagnetic acupuncture for chronic low back pain, the acupoints and procedure have been standardized. In addition, the placebo device will appear and sound, and be used, in the same way as the Whata153, ensuring a double-blind protocol. Moreover, RM-ANOVA will be used to assess not only the reduction in pain at a certain point in time but also differences in the amount of pain reduction over time.

The results of this trial will provide important clinical information about the efficacy and safety of electromagnetic acupuncture, which is expected to cause less discomfort and provide better results than electroacupuncture, for chronic low back pain. We believe that this treatment will relieve pain and improve the quality of life for many patients experiencing chronic low back pain. Because this is a pilot trial, we will analyze the data from various aspects and use statistical methods that will help in providing a foundation for the final clinical trial. If this study is successful, we expect that further studies on electromagnetic acupuncture for other clinical diseases will be designed and performed.

## Author contributions

JHK designed, administrated and supervised the study protocol and critically revised the manuscript. SYO developed the protocol, drafted the manuscript. Both authors have read and approved the final manuscript.

**Conceptualization:** Jae Hui Kang.

**Data curation:** Seo Young OH.

**Formal analysis:** Seo Young OH.

**Funding acquisition:** Jae Hui Kang.

**Investigation:** Seo Young OH, Jae Hui Kang.

**Methodology:** Seo Young OH, Jae Hui Kang.

**Project administration:** Jae Hui Kang.

**Resources:** Seo Young OH.

**Software:** Seo Young OH.

**Supervision:** Jae Hui Kang.

**Validation:** Seo Young OH.

**Visualization:** Seo Young OH.

**Writing – original draft:** Seo Young OH.

**Writing – review & editing:** Jae Hui Kang.
